# Ginsenoside Rb1 Protects Neonatal Rat Cardiomyocytes from Hypoxia/Ischemia Induced Apoptosis and Inhibits Activation of the Mitochondrial Apoptotic Pathway

**DOI:** 10.1155/2014/149195

**Published:** 2014-07-10

**Authors:** Xu Yan, Jinwen Tian, Hongjin Wu, Yuna Liu, Jianxun Ren, Sidao Zheng, Chengying Zhang, Cui Yang, Yang Li, Shengqi Wang

**Affiliations:** ^1^Beijing Haidian Hospital (Haidian Section of Peking University Third Hospital), 29 Zhongguancun Dajie, Haidian District, Beijing 100080, China; ^2^Postdoctoral Workstation of the Zhongguancun Haidian Science Park, NO. 6, Sijiqing Road, Haidian District, Beijing 100195, China; ^3^Institute of Geriatric Cardiology, The General Hospital of People's Liberation Army, 28 Fuxing Road, Beijing 100853, China; ^4^Beijing Hospital of Integrated Traditional Chinese and Western Medicine, Dongjie 3, Yongding Road, Haidian District, Beijing 100039, China; ^5^Beijing Institute of Radiation Medicine, Beijing 100850, China

## Abstract

*Aim*. To investigate the effect of Ginsenoside Rb1 (GS-Rb1) on hypoxia/ischemia (H/I) injury in cardiomyocytes *in vitro* and the mitochondrial apoptotic pathway mediated mechanism. *Methods*. Neonatal rat cardiomyocytes (NRCMs) for the H/I groups were kept in DMEM without glucose and serum, and were placed into a hypoxic jar for 24 h. GS-Rb1 at concentrations from 2.5 to 40 *µ*M was given during hypoxic period for 24 h. NRCMs injury was determined by MTT and lactate dehydrogenase (LDH) leakage assay. Cell apoptosis, ROS accumulation, and mitochondrial membrane potential (MMP) were assessed by flow cytometry. Cytosolic translocation of mitochondrial cytochrome c and Bcl-2 family proteins were determined by Western blot. Caspase-3 and caspase-9 activities were determined by the assay kit. *Results*. GS-Rb1 significantly reduced cell death and LDH leakage induced by H/I. It also reduced H/I induced NRCMs apoptosis induced by H/I, in accordance with a minimal reactive oxygen species (ROS) burst. Moreover, GS-Rb1 markedly decreased the translocation of cytochrome c from the mitochondria to the cytosol, increased the Bcl-2/ Bax ratio, and preserved mitochondrial transmembrane potential (ΔΨm). Its administration also inhibited activities of caspase-9 and caspase-3. *Conclusion*. Administration of GS-Rb1 during H/I *in vitro* is involved in cardioprotection by inhibiting apoptosis, which may be due to inhibition of the mitochondrial apoptotic pathway.

## 1. Introduction

Myocardial ischemic injury resulting from severe impairment of the coronary blood supply is a severe stress that leads to the loss of cardiomyocytes by apoptosis and necrosis. Activation of the mitochondrial apoptotic pathway is seen as a common cause of cell death in numerous cardiac diseases [1, 2]. The mitochondrial apoptotic pathway is characterized by mitochondrial dysfunction with release of caspase activators, including mitochondrial permeability transition pore (mPTP) opening, loss of mitochondrial transmembrane potential (ΔΨm), cytochrome c release, and changes in Bcl2/Bax ratio, which are followed by activation of caspase-9 and caspase-3 [3, 4]. Specific inhibition of the mitochondrial apoptotic pathway could protect cardiomyocytes from injuries, which implies that mitochondria could be a critical site for intervention [5, 6].

Ginseng, the root of* Panax ginseng* Meyer, has been widely used for more than 2,000 years in China. [7]. Its beneficial effects act on the endocrine, immune, central nervous and, especially, the cardiovascular systems [8]. Ginsenoside Rb1 (GS-Rb1), the major pharmacological extract, is one of the most important active compounds of ginseng, with extensive evidence of its cardioprotective properties. GS-Rb1 can protect cardiomyocytes from H_2_O_2_ induced oxidative injury by suppressing JNK activation [9]. It can exert its cardioprotective effect against ischemia reperfusion (MI/R) injury in diabetic rats by activation of the phosphatidylinositol 3-kinase (PI3 K)/Akt pathway [10], and GS-Rb1 preconditioning can enhance eNOS expression and attenuate myocardial ischemia/reperfusion injury in diabetic rats [11]. However, the specific mechanisms of cardioprotective properties of GS-Rb1 in a hypoxic environment* in vitro* and the changes of the anti- and proapoptotic proteins involved need clarification.

In the present research, we have investigated the protective effect of GS-Rb1 and its impact on the mitochondrial apoptotic pathway, in neonatal rat cardiomyocytes (NRCMs) exposed to hypoxic/ischemic damage. It had antiapoptotic activity under the hypoxic/ischemic conditions* in vitro* and inhibited activation of the mitochondrial apoptotic pathway.

## 2. Materials and Methods

### 2.1. Materials

GS-Rb1 (catalog number #110704), purchased from National Institutes for food and drug Control, was dissolved in phosphate-buffered saline (PBS) to create a stock solution for subsequent dilution. Dulbecco's Modified Eagle Medium (DMEM) and fetal bovine serum (FBS) were obtained from Gibco (Grand Island, NY, USA). DMSO and 3-(4, 5-dimethyl-thiazol-2-yl)-2, 5-diphenyltetrazoliumbromide (MTT) were obtained from Sigma (St.Louis, MO, USA). Cell Lysis Buffer for Western blotting and IP and Enhanced BCA Protein Assay Kit were obtained from Beyotime (Haimen, China). The primary antibodies against Bcl-2 (catalog number #2870), Bax (catalog number #2772), cytochrome c (catalog number #4272), GAPDH (catalog number #2118), and horseradish peroxidase (HRP)-conjugated secondary antibodies were obtained from Cell Signaling Technology (Danvers, MA, USA). Enhanced chemiluminescence kit was obtained from Millipore (Billerica, MA, USA).

### 2.2. Culture of Neonatal Rat Cardiomyocytes

Primary cultures of NRCMs from 12 to 24 h old Sprague Dawley rats (Vital River Laboratories, Beijing, China) were prepared by means of gentle serial trypsinization as described before with slight modification [12]. All experiments were approved by the Beijing Ethics Committee for the Use of Experimental Animals. The NRCMs were plated in collagen-coated 96- or 6-well plates and maintained at 37°C in a 5% CO_2_/95% air humidified incubator in DMEM containing 10% (v/v) fetal bovine serum, 100 U/mL penicillin, and 100 mg/mL streptomycin. The following experiments used spontaneously beating cardiomyocytes 48–72 h after plating.

### 2.3. Hypoxia/Ischemia Treatment

For hypoxic/ischemic protocol, hypoxia was achieved by using the MGC AnaeroPack System in a AnaeroPack jar (Mitsubishi Gas Chemical Co., Tokyo, Japan), which was capable of depleting the concentration of O_2_ down to 10% in 2 h. Ischemic condition was achieved by replacing culture medium with DMEM without glucose (Gibco, Grand Island, USA) and serum. NRCMs with or without GS-Rb1 were placed in the AnaeroPack jar and then put into a 37°C incubator for 24 h. Control plates were kept in normoxic conditions for the corresponding times.

### 2.4. MTT Assay

NRCMs viability was determined using the MTT assay. Cardiomyocytes were plated on 96-well dishes at 2 × 10^4^ cells/well. MTT at 5 mg/mL was added to each well immediately after 24 h of hypoxia/ischemia. Plates were incubated for 4 h at 37°C. The medium was aspirated from each well and 100 *μ*L of DMSO was added to dissolve the formazan crystals. The optical density of each well was read at 492 nm using a Microplate Reader (Bio-Rad, Hercules, CA). Results are given as percentages of the control group taken as 100%.

### 2.5. Assay of LDH Activity

The extent of cellular injury was monitored by LDH leakage. Culture medium (120 *μ*L) was taken to measure LDH activity using a commercial kit with a spectrophotometer (JianCheng Bioengineering Institute, Nanjing, China).

### 2.6. Apoptosis Assay by Annexin V/PI Staining

NRCMs with different concentration of GS-Rb1 exposed to hypoxia/ischemia conditions were harvested and washed with PBS. The percentage of normal nonapoptotic cells and apoptotic cells was measured by double supravital staining with Annexin-V and PI, using an Annexin V-FITC Apoptosis Detection kit (KeyGen, Nanjing, China). Flow cytometric analysis used a Cytomics FC500 flow cytometer with CXP software (Beckman Coulter, Fullerton, USA), the operator being blind of the group assignment.

### 2.7. Fluorescent Measurement of Intracellular ROS

Determination of ROS concentration was based on the oxidation of 2,7-dichlorodihydrofluorescein (DCFH) (JianCheng Bioengineering Institute, Nanjing, China). In brief, the cells were collected after hypoxia/ischemia and washed with DMEM without FBS and incubated with DCFH-DA at 37°C for 20 min. Dichlorofluorescein (DCF) fluorescence intensity was detected at 488 nm excitation and 525 nm emission by flow cytometry and the mean fluorescence intensity used to represent the amount of ROS.

### 2.8. Determination of ΔΨm

The mitochondrial transmembrane potential (ΔΨm) was estimated by monitoring fluorescence aggregates of 5,5′,6,6′-tetrachloro-1,1′,3,3′-tetraethyl benzimidazolo carbocyanine iodide (JC-1), (Beyotime, Haimen, China). Briefly, NRCMs were collected and incubated at 37°C for 30 min with JC-1 (1×), washed twice with PBS for detection of the fluorescent ratio by flow cytometry. JC-1 fluorescence was measured to assess the emission shift from green (530 nm) to red (590 nm) using 488 nm excitation. Data are given as the relative ratio of green to red fluorescence intensity, indicating the level of depolarization of the mitochondrial membrane potential.

### 2.9. Isolation of Mitochondria

Cytochrome c release from mitochondria was measured by Western blotting. The preparation of the mitochondrial and cytosolic protein fractions was done with a Cell Mitochondria Isolation Kit (Beyotime, Haimen, China). Briefly, cells were collected and resuspended in mitochondrial isolation reagent containing PMSF and protease inhibitors, centrifuged at 600 ×g for 10 min at 4°C. The supernatant was transferred to a fresh 1.5 mL microcentrifuge tube and centrifuged at 11,000 ×g for 10 min at 4°C. The supernatant was collected as the cytosolic fraction and the pellet was resuspended in 0.1 mL mitochondrial lysis buffer, which was kept as the mitochondrial fraction.

### 2.10. Western Blotting

The mitochondria and NRCMs with under hypoxic and ischemic conditions treated with different concentrations of GS-Rb1 for 24 h were harvested and lysed with Cell Lysis Buffer for Western blotting and IP. Protein concentration was measured using a BCA Protein Assay Kit. Equal amounts of sample lysate were separated by sodium dodecyl sulfate polyacrylamide gel electrophoresis (SDS-PAGE) and transferred by electroblotting to a nitrocellulose membrane (Millipore). The membrane was blocked with 5% nonfat milk in TBST buffer (20 mM Tris-HCl, pH 7.4, 150 mM NaCl and 0.1% Tween 20) overnight at 4°C. The membrane was incubated with specific primary antibodies for 2 h and an IgG HRP conjugated specific secondary antibody for 1 h at room temperature. The signals were visualized with an enhanced chemiluminescence (ECL) kit (Pierce) on Syngene G: BOX Chemi gel documentation system (Syngene, Cambridge, UK). Densitometric values were normalized using GAPDH in each group as an internal control.

### 2.11. *In Vitro* Caspase-3 and Caspase-9 Activity Assay

Caspase activities was measured by Caspase-3 and Caspase-9 Activity Assay Kit (Beyotime, Haimen, China). Briefly, NRCMs lysates were prepared after treatment with different dose of GS-Rb1 for 24 h under hypoxia/ischemia conditions. Assays were performed on 96-well microtitre plates by incubating 10 *μ*L protein cell lysate per sample in 80 *μ*L reaction buffer containing 10 *μ*L substrate (Asp-Glu-Val-Asp (DEVD)-p-nitroaniline (pNA) for caspase-3, and Leu-Glu-His-Asp (LEHD)-pNA for caspase-9, 2 mM). Lysates were incubated at 37°C for 4–6 h. Samples were measured with a Microplate Reader (Bio-Rad, Hercules, CA) at an absorbance of 405 nm. Caspase activity was expressed as the percentage relative to the control group.

### 2.12. Statistical Analysis

Data are expressed as the mean ± SD of at least three independent experiments. Group results were analysed for variance using ANOVA. Two groups were compared by Student's *t*-test. All analyses used GraphPad Prism 5.0 software. *P* < 0.05 was taken as statistically significant.

## 3. Results

### 3.1. Effect of GS-Rb1 on the Viability of NRCMs

**Figure 1:**
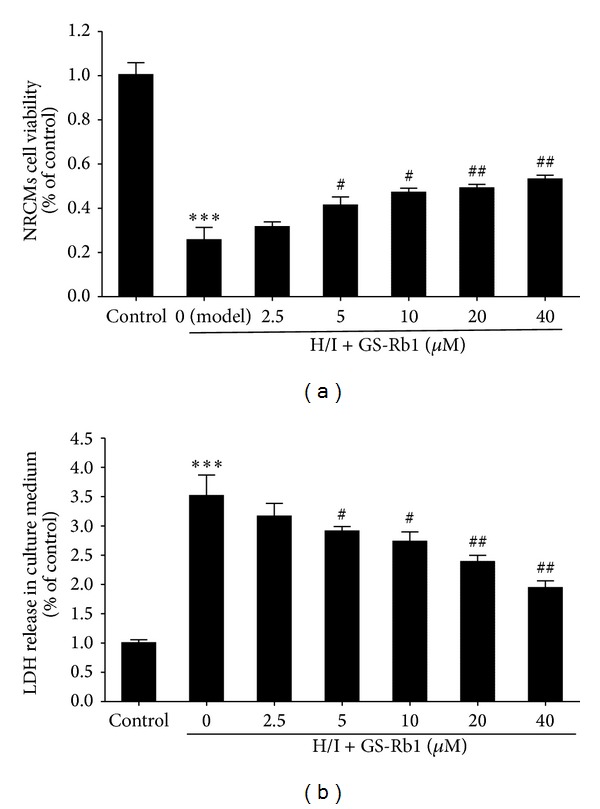
Protective effect of GS-Rb1 on H/I induced NRCMs death. NRCMs were copretreated with or without GS-Rb1 during H/I condition for 24 h. (a) Cell viability determined by MTT assay. (b) LDH release determined by the LDH activity kit. Error bars represent mean ± SD. ^∗∗∗^ *P* < 0.001 versus control; ^#^ *P* < 0.05 and ^##^ *P* < 0.01 versus H/I group (*n* = 3).

The effect of GS-Rb1 on NRCMs viability was examined by the MTT assay (Figure 1(a)). Those given H/I accounted for 25% of the normal control cells (*P* < 0.001). Compared with the control group, GS-Rb1 from 2.5 to 40 *μ*M incubation increased viability from 31.7% ± 1.3% to 53.3% ± 1.7%. GS-Rb1 alone had no effect on cell viability.

### 3.2. Effect of GS-Rb1 on the LDH Leakage Induced by H/I

H/I treatment increased LDH leakage from NRCMs by 3.5 ± 0.2-fold in comparison with the control group. GS-Rb1 incubation significantly reduced leakage induced to 1.9 ± 0.12-fold (Figure 1(b)).

### 3.3. GS-Rb1 Reduced Apoptotic Cells Induced by H/I in NRCMs

**Figure 2:**
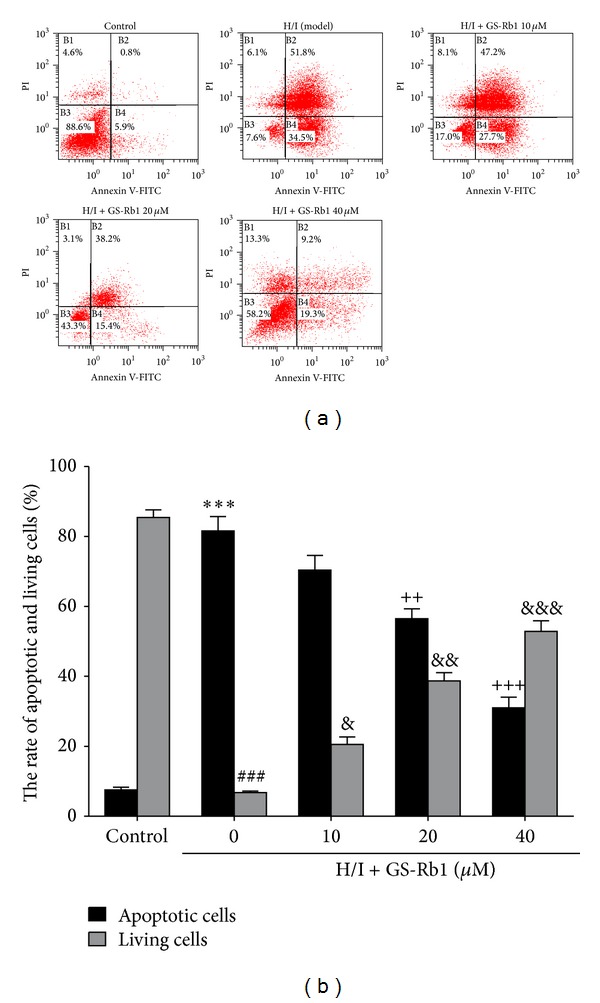
Flow cytometry analysis of GS-Rb1 on cell death induced by H/I. (a) NRCMs were cotreated with or without GS-Rb1 (10, 20, and 40 *μ*M) during H/I, for 24 h, and stained with Annexin V-fitc/PI. (b) Quantification of the percent of apoptotic and living cells in each group. Error bars represent mean ± SD. ^∗∗∗^ *P* < 0.001 versus apoptotic cells in control group; ^###^ *P* < 0.001 versus living cells in control group; ^++^ *P* < 0.01 and ^+++^ *P* < 0.001 versus apoptotic cells in H/I group; ^&^ *P* < 0.05, ^&&^ *P* < 0.01, and ^&&&^ *P* < 0.001 versus living cells in H/I group (*n* = 3).

To examine the effect of GS-Rb1 on the cell death induced by H/I, Annexin V-fitc/propidium iodide (PI) double-staining assay of cells was analyzed by flow cytometry (Figures 2(a) and 2(b)). The percentage of apoptotic cells (including early and late apoptotic cells) markedly increased in H/I group compared to the control group. With GS-Rb1, apoptosis accounted for 70.4 ± 2.4, 56.5 ± 1.7, and 31.0 ± 1.8% at 10, 20, and 40 *μ*M GS-Rb1 for 24 h, respectively, with the surviving cells increasing from 6.8 ± 0.4 to 52.8 ± 3.1%.

### 3.4. Effect of GS-Rb1 on the Intracellular ROS Level in NRCMs

**Figure 3:**
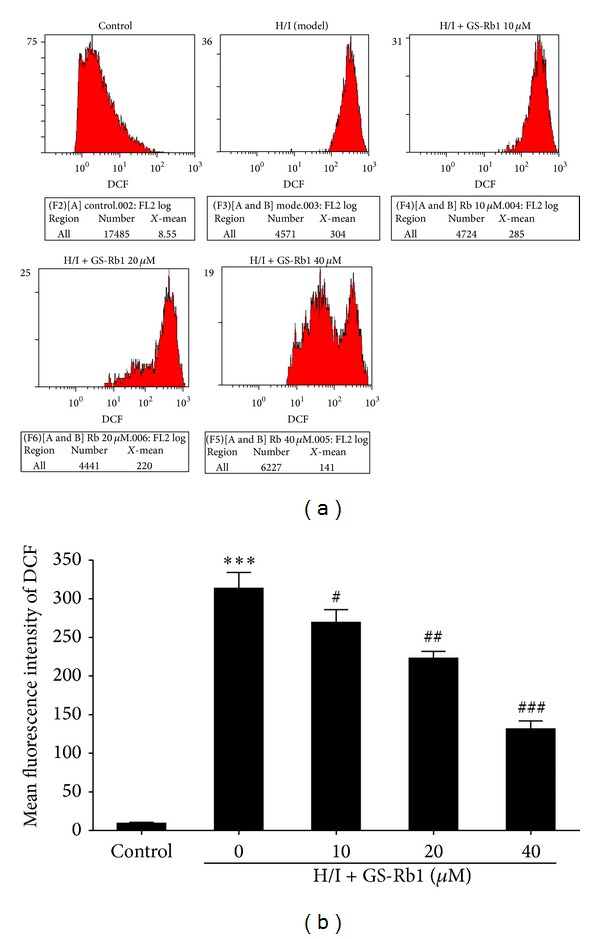
Flow cytometry analysis of GS-Rb1 on intracellular ROS induced by H/I. (a) NRCMs were cotreated with or without GS-Rb1 (10, 20, and 40 *μ*M) during H/I for 24 h. The intracellular ROS level was measured by the fluorescent probe DCFH-DA. (b) Analysis of the mean fluorescence intensity of each group. Error bars represent mean ± SD. ^∗∗∗^ *P* < 0.001 versus control, ^#^ *P* < 0.05, ^##^ *P* < 0.01, and ^###^ *P* < 0.001 versus H/I group (*n* = 3).

Cells in different groups were harvested and stained with fluorescent probe DCFH before being analyzed by flow cytometry. H/I treatment significantly increased the intracellular level of ROS, with the fluorescence intensity increasing from 9.3 ± 0.9 to 313.3 ± 11.9 (Figures 3(a) and 3(b)). Cotreatment with GS-Rb1 significantly inhibited the raised intracellular concentration of ROS induced by H/I. These results indicate that the cardioprotective effect of GS-Rb1 from H/I injury is associated with the inhibition of cellular ROS.

### 3.5. Effect of GS-Rb1 on ΔΨm in NRCMs

**Figure 4:**
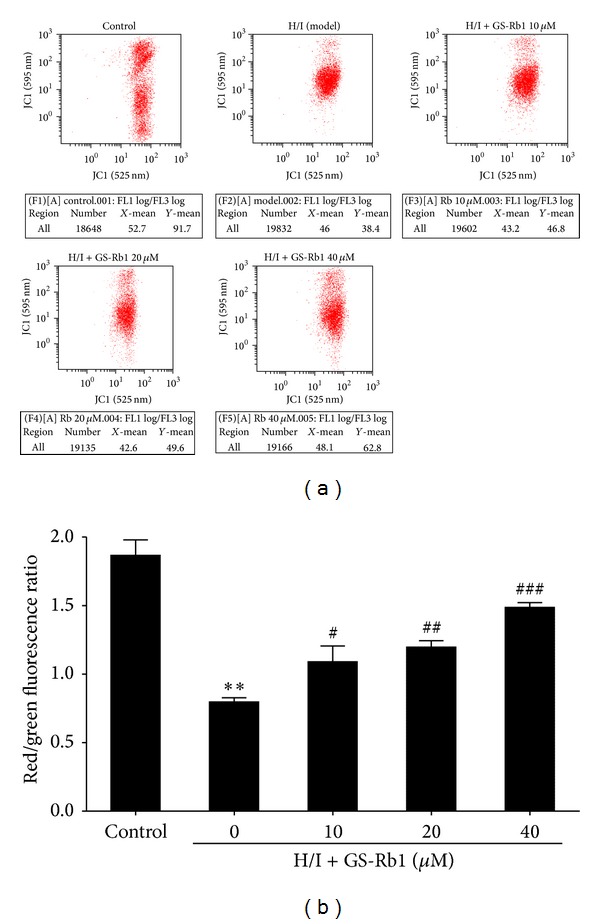
Flow cytometry analysis of GS-Rb1 on mitochondrial membrane potential (ΔΨm) induced by H/I. (a) NRCMs were cotreated with or without GS-Rb1 (10, 20, and 40 *μ*M) during H/I for 24 h. ΔΨm was measured by JC-1 and analyzed by flow cytometry. (b) Analysis of the red/green fluorescence ratio of each group. Error bars represent mean ± SD. ^∗∗∗^ *P* < 0.001 versus control, ^#^ *P* < 0.05, ^##^ *P* < 0.01, and ^###^ *P* < 0.001 versus H/I group, (*n* = 3).

Loss of ΔΨm indicates mitochondrial dysfunction that ultimately leads to apoptosis. JC-1 staining was carried out before flow cytometric analysis. NRCMs exposed to H/I condition for 24 h resulted in dissipation of ΔΨm compared with the control group (Figures 4(a) and 4(b)), seen as increased green fluorescence. Cotreatment with different concentrations of GS-Rb1 moderated the dissipation of ΔΨm, indicating its protective effect. The ratio of green and red fluorescence was used to demonstrate the mitochondrial membrane potential (ΔΨm) change induced by H/I treatment and the protective effect of GS-Rb1. In the control group, JC-1 aggregated in mitochondria with a ratio of 1.87 ± 0.065, that of the H/I group being 0.797 ± 0.018. The groups cotreated with 10, 20, and 40 *μ*M GS-Rb1 showed increases of 1.090 ± 0.067 to 1.487 ± 0.020, which suggests that the mitochondria-mediated pathway was involved in the cardioprotective effect of GS-Rb1 on H/I injuries.

### 3.6. Effects of GS-Rb1 on the Release of Cytochrome c

**Figure 5:**
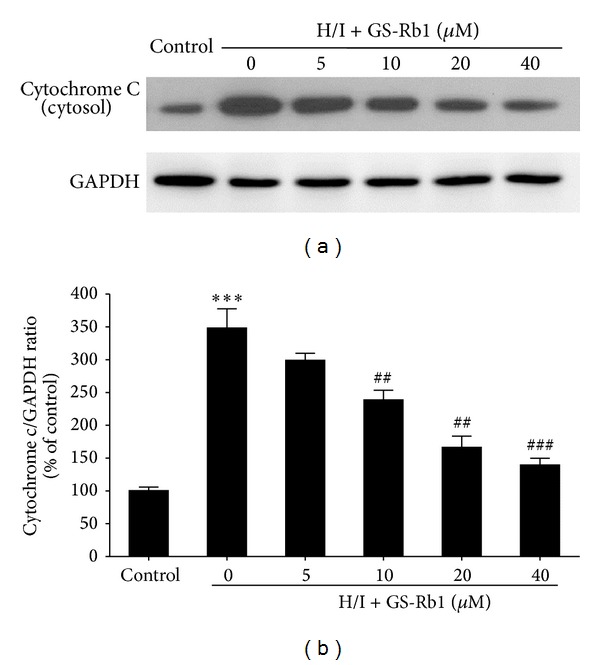
Effect of GS-Rb1 on cytosolic cytochrome c level change induced by H/I. NRCMs were cotreated with or without GS-Rb1 (5, 10, 20, and 40 *μ*M) during H/I for 24 h. (a) Cytochrome c was measured by Western blot. (b) Quantitative data was analysed for each group. Error bars represent mean ± SD. ^∗∗∗^ *P* < 0.001 versus control; ^##^ *P* < 0.01 and ^###^ *P* < 0.001 versus H/I group (*n* = 3).

The release of cytochrome c from mitochondria into cytosol is one of the early events leading to apoptosis. Increased mitochondrial membrane permeability was confirmed by the translocation of cytochrome c from the mitochondria to the cytosol. Few cytochromes c in the cytoplasm were found in the control group (Figures 5(a) and 5(b)) whereas an obvious increase of cytochrome c in cytosol occurred in H/I treated cells (348.2 ± 16.9). Compared with the H/I group, cytochrome c in cytosol in the GS-Rb1 treatment group decreased from 298.9 ± 6.3 to 139.6 ± 5.9. Gs-Rb1 cotreatment significantly inhibited H/I-induced cytochrome c release into the cytoplasm, which shows again that GS-Rb1 acts on the mitochondria mediated pathway in its cardioprotective effect.

### 3.7. Effect of GS-Rb1 on the Expression of Bax and Bcl-2 following H/R Injury

**Figure 6:**
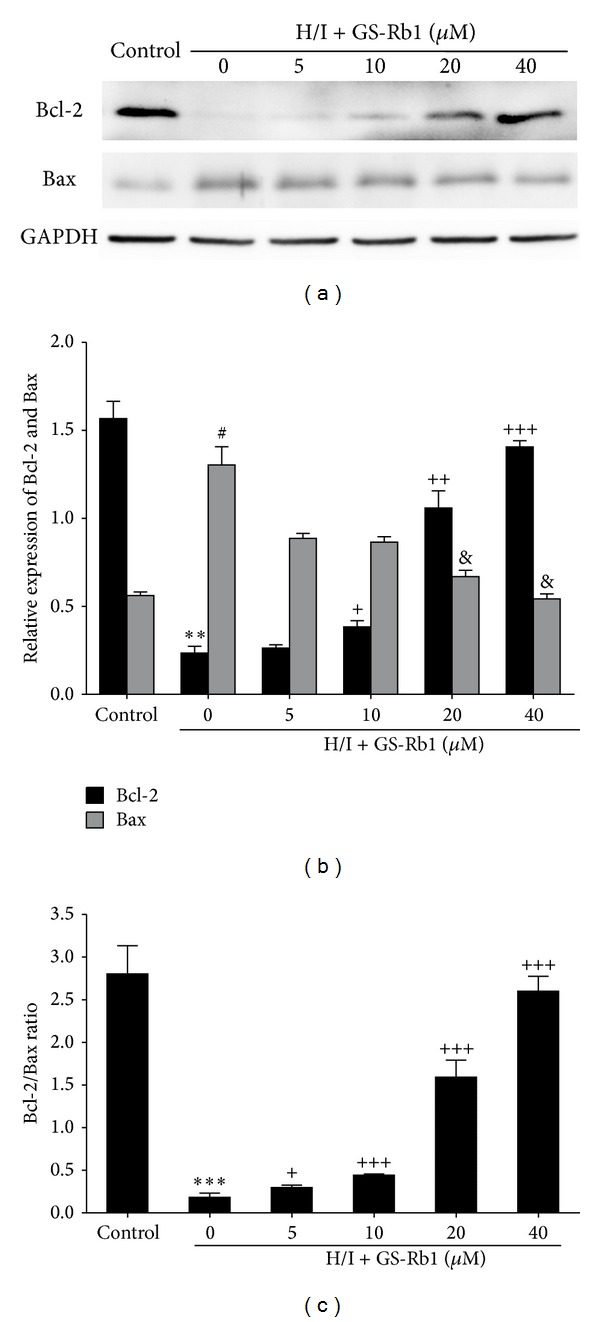
Effect of GS-Rb1 on Bcl-2 and Bax changes induced by H/I. NRCMs were cotreated with or without GS-Rb1 (5, 10, 20, and 40 *μ*M) during H/I for 24 h. (a) The protein levels of Bcl-2 and Bax were quantified by Western blot. (b) Quantitative data analysis for each group. (c) Bcl-2/Bax protein expression ratio in each group. Error bars represent mean ± SD. ^∗∗∗^ *P* < 0.001 versus Bcl-2 in control group; ^##^ *P* < 0.01 versus Bax in control group; ^+^ *P* < 0.05, ^++^ *P* < 0.01, and ^+++^ *P* < 0.001 versus Bcl-2 in H/I group; ^&^ *P* < 0.05 and ^&&^ *P* < 0.01 versus Bax in H/I group.

Bcl-2 family proteins have a function in mitochondria mediated apoptosis. Expression of antiapoptotic Bcl-2 and proapoptotic Bax proteins were analyzed by Western blot after H/I and GS-Rb1 treatment. Compared with the control group, H/I significantly increased Bax expression and decreased Bcl-2. The ratio of Bcl-2 to Bax decreased over 10-fold. With GS-Rb1 cotreatment, increased levels of Bcl-2 and a marked reduction in Bax were observed in a dose-dependent manner (Figures 6(a), 6(b), and 6(c)). Thus, GS-Rb1 inhibits apoptosis induced by H/I by upregulating the ratio of Bcl-2 to Bax.

### 3.8. Effect of GS-Rb1 on the Activation of Caspase 3 and Caspase 9

**Figure 7:**
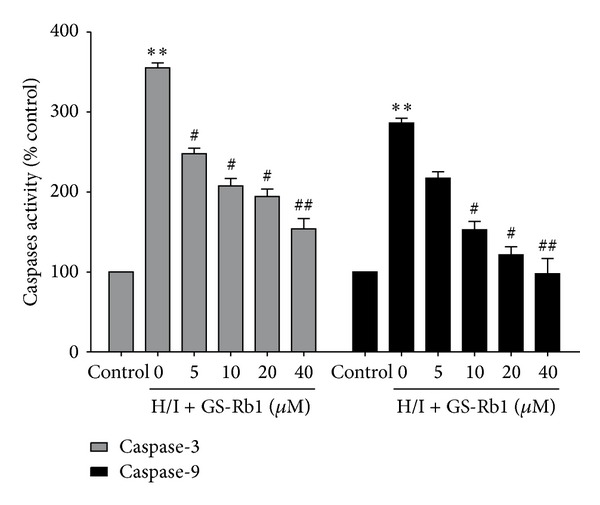
Effect of GS-Rb1 on caspase-3 and caspase-9 activities induced by H/I. NRCMs were cotreated with or without GS-Rb1 (5, 10, 20, and 40 *μ*M) during H/I for 24 h. NRCMs were lysed and caspase-3 and caspase-9 activities were analyzed. Error bars represent mean ± SD. ^∗∗^ *P* < 0.01 versus control group; ^#^ *P* < 0.05 and ^##^ *P* < 0.01 versus H/I group (*n* = 3).

Activation of caspase-3 and caspase-9 is a key irreversible point in mitochondria mediated apoptosis. When compared with the controls (Figure 7), H/I significantly increased the activity of caspase-3 and caspase-9 by 355.3 ± 6.0 and 286.2 ± 6.1%, respectively. When cotreated with different doses of GS-Rb1, activities of caspase-3 and caspase-9 were attenuated from 247.8 ± 7.0 to 153.7 ± 13.1 and 217.3 ± 7.8% to 97.8 ± 19.0%, respectively. These results indicated that GS-Rb1 could inhibit apoptosis induced by H/I by attenuating caspase-3 and caspase-9 activity.

## 4. Discussion

GS-Rb1 is one of the most important active compounds of ginseng, which has multiple pharmacological actions, including antifatigue [13], reducing fatty liver [14], antiobesity [15], antiskin aging [16], protective effects of central nervous system [17], retinal ganglion [18], lung [19], liver [20], and renal injury [21]. GS-Rb1 has an established cardiovascular protective effect. The protective effects of GS-Rb1 on neonatal rat cardiomyocytes (NRCMs) have been shown here by MTT combined with LDH leakage assay in increasing the viability of NRCMs when subjected in the H/I conditions in a dose-dependent manner. Flow cytometric analysis showed that cardioprotection in response to GS-Rb1 was due to inhibition of apoptosis, cellular ROS production, and prevention of ΔΨm loss. Our novel finding is that GS-Rb1 significantly inhibited the release of cytochrome c from mitochondria into cytosol and maintained the ratio of Bcl-2 to Bax. It also regulated downstream both caspase-3 and caspase-9 activity.

Apoptosis is heavily involved in the development and progression of cardiovascular disease [22]. Blocking it can prevent the loss of contractile cells and minimize cardiac injury [23]. H/I induces NRCMs apoptosis (including early and late apoptosis). With H/I alone, GS-Rb1 attenuated cardiomyocyte apoptosis from 81.6 to 31.0%, while living cells rose from 6.78 to 52.8%. Therefore, cotreatment with GS-Rb1 mitigates H/I-induced apoptosis of cardiomyocytes.

Ischemia injuries provoke ROS generation in cardiomyocytes [24], and these have a secondary messenger function because of their ability to influence MMP and mitochondrial function, thereby activating the caspase cascade [25]. When ROSs reach a threshold level, opening of transition pores is triggered, which decreases ΔΨm and induces the side leakage of caspase-activating proteins, that is, cytochrome c to the cytosol [26, 27]. GS-Rb1 is known to be protective to neonatal rat cardiomyocytes treated with from CoCl_2_ to induce hypoxic injuries by inhibiting GSK-3*β*-mediated mPTP opening [28]. Our results demonstrate that H/I induce ΔΨm loss and ROS accumulation and that GS-Rb1 significantly inhibits ΔΨm depolarization and ROS production. These results suggest that the cardioprotective effects of GS-Rb1 might be related to the inhibition of ROS rather than the generation and preservation of the mitochondria.

It has been demonstrated that mitochondrial permeability and release of the apoptosome could be controlled by Bcl-2 family [29]. An increase of proapoptosis proteins (Bax and Bid) and/or a decrease in antiapoptosis proteins (Bcl-2 and Bcl-xL) could lead to a decrease in mitochondrial membrane potential and an opening of mitochondrial permeability transition pores, leading to cytochrome c release from mitochondria into cytosol [30]. The current research showed that ratio of Bcl-2 to Bax was increased by GS-Rb1 treatment following H/I, which suggested that Bcl-2 family was involved in the cardioprotection of GS-Rb1 in H/I.

Mitochondria play an important role in cardiomyocytes injuries by releasing cytochrome c and other apoptogenic proteins into the cytosol [31]. Cytochrome c in the cytosol forms a complex that is composed of apoptosis-activating factor-1 (Apaf-1), which recruits and activates the death effector caspase-9 [32]. The activated caspase-9 subsequently activates caspase-3, which in turn activates the early apoptosis process [33]. Our results showed that GS-Rb1 significantly inhibited mitochondrial cytochrome c release and caspase-3 and caspase-9 activity following H/I, which suggested that cytochrome c release and the mitochondrial dependent pathway were involved in the cardioprotection of GS-Rb1 in H/I.

## 5. Conclusion

In conclusion, GS-Rb1 exerts a protective effect in hypoxia/ischemia-induced cell death, which may underlie the inhibition of the mitochondrial apoptotic pathway. The cardioprotective effect seems to act by decreasing cellular ROS production, restoring ΔΨm, attenuating H/I-mediated mitochondrial cytochrome c release, reducing the ratio of Bax to Bcl-2, and thereby affecting caspase-3 and caspase-9 activity. Further work will be required to understand the beneficial role and the effect on other organs of GS-Rb1 in postischemic injury* in vivo*, with the hope that these benefits can be translated to clinical interventions in the treatment of ischemic heart diseases.

## References

[1] Sharov VG, Todor A, Khanal S, Imai M, Sabbah HN (2007). Cyclosporine A attenuates mitochondrial permeability transition and improves mitochondrial respiratory function in cardiomyocytes isolated from dogs with heart failure. *Journal of Molecular and Cellular Cardiology*.

[2] Li Q, Zhou L, Gao G, Jiao J, Li P (2012). Mitochondrial network in the heart. *Protein and Cell*.

[3] Bouchier-Hayes L, Lartigue L, Newmeyer DD (2005). Mitochondria: pharmacological manipulation of cell death. *The Journal of Clinical Investigation*.

[4] Schonhoff CM, Gaston B, Mannick JB (2003). Nitrosylation of cytochrome c during apoptosis. *Journal of Biological Chemistry*.

[5] Guo C, Zeng X, Song J (2012). A soluble receptor for advanced glycation end-products inhibits hypoxia/reoxygenation-induced apoptosis in rat cardiomyocytes via the mitochondrial pathway. *International Journal of Molecular Sciences*.

[6] Huang H, Wu K, You Q, Huang R, Li S (2013). Naringin inhibits high glucose-induced cardiomyocyte apoptosis by attenuating mitochondrial dysfunction and modulating the activation of the p38 signaling pathway. *International Journal of Molecular Medicine*.

[7] Xu ML, Kim H-J, Choi Y-R, Kim H-J (2012). Intake of Korean red ginseng extract and saponin enhances the protection conferred by vaccination with inactivated influenza A virus. *Journal of Ginseng Research*.

[8] Jia L, Zhao Y, Liang X (2009). Current evaluation of the millennium phytomedicine - Ginseng (II): Collected chemical entities, modern pharmacology, and clinical applications emanated from traditional chinese medicine. *Current Medicinal Chemistry*.

[9] Li J, Shao ZH, Xie JZ (2012). The effects of ginsenoside Rb1 on JNK in oxidative injury in cardiomyocytes. *Archives of Pharmacal Research*.

[10] Wu Y, Xia ZY, Dou JJ (2011). Protective effect of ginsenoside Rb1 against myocardial ischemia/reperfusion injury in streptozotocin-induced diabetic rats. *Molecular Biology Reports*.

[11] Xia R, Zhao B, Wu Y (2011). Ginsenoside Rb1 preconditioning enhances eNOS expression and attenuates myocardial ischemia/reperfusion injury in diabetic rats. *Journal of Biomedicine and Biotechnology*.

[12] Simpson P, Savion S (1982). Differentiation of rat myocytes in single cell cultures with and without proliferating nonmyocardial cells. Cross-striations, ultrastructure, and chronotropic response to isoproterenol. *Circulation Research*.

[13] Tan S, Zhou F, Li N (2013). Anti-fatigue effect of ginsenoside Rb1 on postoperative fatigue syndrome induced by major small intestinal resection in rat. *Biological & Pharmaceutical Bulletin*.

[14] Shen L, Xiong Y, Wang DQ (2013). Ginsenoside Rb1 reduces fatty liver by activating AMP-activated protein kinase in obese rats. *Journal of Lipid Research*.

[15] Lin N, Cai D, Jin D, Chen Y, Shi J (2014). Ginseng panaxoside Rb1 reduces body weight in diet-induced obese mice. *Cell Biochemistry and Biophysics*.

[16] Kang TH, Park HM, Kim Y (2009). Effects of red ginseng extract on UVB irradiation-induced skin aging in hairless mice. *Journal of Ethnopharmacology*.

[17] Liu D, Zhang H, Gu W, Liu Y, Zhang M (2013). Neuroprotective effects of ginsenoside rb1 on high glucose-induced neurotoxicity in primary cultured rat hippocampal neurons. *PLoS ONE*.

[18] Liu Z, Chen J, Huang W, Zeng Z, Yang Y, Zhu B (2013). Ginsenoside Rb1 protects rat retinal ganglion cells against hypoxia and oxidative stress. *Molecular Medicine Reports*.

[19] Wang J, Qiao L, Li S, Yang G (2013). Protective effect of ginsenoside rb1 against lung injury induced by intestinal ischemia-reperfusion in rats. *Molecules*.

[20] Wang J, Qiao L, Li Y, Yang G (2008). Ginsenoside Rb1 attenuates intestinal ischemia-reperfusion-induced liver injury by inhibiting NF-*κ*B activation. *Experimental and Molecular Medicine*.

[21] Sun Q, Meng Q, Jiang Y, Xia Z (2012). Ginsenoside Rb1 attenuates intestinal ischemia reperfusion induced renal injury by activating Nrf2/ARE pathway. *Molecules*.

[22] Lee Y, Gustafsson ÅB (2009). Role of apoptosis in cardiovascular disease. *Apoptosis*.

[23] Song J, Teng X, Cai Y, Tang C, Qi Y (2009). Activation of Akt/GSK-3*β* signaling pathway is involved in intermedin1–53 protection against myocardial apoptosis induced by ischemia/reperfusion. *Apoptosis*.

[24] Cadenas S, Aragonés J, Landázuri MO (2010). Mitochondrial reprogramming through cardiac oxygen sensors in ischaemic heart disease. *Cardiovascular Research*.

[25] Ly JD, Grubb DR, Lawen A (2003). The mitochondrial membrane potential (*δψ*m) in apoptosis; an update. *Apoptosis*.

[26] Zorov DB, Juhaszova M, Sollott SJ (2006). Mitochondrial ROS-induced ROS release: an update and review. *Biochimica et Biophysica Acta*.

[27] Circu ML, Aw TY (2010). Reactive oxygen species, cellular redox systems, and apoptosis. *Free Radical Biology and Medicine*.

[28] Kong HL, Li ZQ, Zhao YJ (2010). Ginsenoside Rb1 protects cardiomyocytes against CoCl2-induced apoptosis in neonatal rats by inhibiting mitochondria permeability transition pore opening. *Acta Pharmacologica Sinica*.

[29] Yang E, Korsmeyer SJ (1996). Molecular thanatopsis: a discourse on the BCL2 family and cell death. *Blood*.

[30] van Loo G, Saelens X, van Gurp M, MacFarlane M, Martin SJ, Vandenabeele P (2002). The role of mitochondrial factors in apoptosis: a Russian roulette with more than one bullet. *Cell Death & Differentiation*.

[31] Honda HM, Ping P (2006). Mitochondrial permeability transition in cardiac cell injury and death. *Cardiovascular Drugs and Therapy*.

[32] Yuanming H, Benedict MA, Ding L, Núñez G (1999). Role of cytochrome c and dATP/ATP hydrolysis in Apaf-1-mediated caspase-9 activation and apoptosis. *EMBO Journal*.

[33] Nishida K, Yamaguchi O, Otsu K (2008). Crosstalk between autophagy and apoptosis in heart disease. *Circulation Research*.

